# Drug Resistance and Comorbidities in the Treatment of Pulmonary Tuberculosis: A Multicenter Retrospective Cohort Study

**DOI:** 10.3390/antibiotics14100986

**Published:** 2025-10-01

**Authors:** Nikolay N. Osipov, Dmitry Spelnikov, Ekaterina Belyaeva, Anastasia Kulpina, Mikhail Nazarenko, Gudkin Mikhail, Nikolay Yu. Nikolenko, Dmitry Kudlay, Anna Starshinova

**Affiliations:** 1 Department of Mathematics and Computer Science, St. Petersburg State University, St. Petersburg 199034, Russia; nicknick@pdmi.ras.ru (N.N.O.); spelnikov_d@spg.ru (D.S.); ekaterina_83@bk.ru (E.B.); asya.starshinova@mail.ru (A.K.); 2 St. Petersburg Department of Steklov Mathematical Institute, St. Petersburg 191023, Russia; 3 Almazov National Medical Research Centre, St. Petersburg 197341, Russia; 4 St. Petersburg Pushkin Antituberculosis Dispensary, St. Petersburg 196602, Russia; drpulmone@yandex.ru; 5 Interdistrict Petrogradsko-Primorsky Anti-Tuberculosis Dispensary N. 3, St. Petersburg 197343, Russia; ptd3@spb.ru; 6 Moscow Research and Clinical Center for Tuberculosis Control, Moscow 107014, Russia; nynikolenko@ya.ru; 7 Department of Pharmacology, Institute of Pharmacy, M. Sechenov First Moscow State Medical University, Moscow 119991, Russia; kudlay_d_a@staff.sechenov.ru; 8 Department of Pharmacognosy and Industrial Pharmacy, Faculty of Fundamental Medicine, Lomonosov Moscow State University, Moscow 119991, Russia; 9 Institute of Immunology FMBA of Russia, Moscow 115522, Russia

**Keywords:** anti-tuberculosis drugs, drug-resistant tuberculosis, comorbidity, extensive drug resistance, XDR-TB, bedaquiline, thioamides, heard disease, cardiotoxicity

## Abstract

Tuberculosis (TB) probably returned to being the world’s leading cause of death from a single infectious agent after three years during which it was replaced by COVID-19. Currently, there are two major, closely related challenges in TB treatment: a large number of cases of drug-resistant TB, as well as cases complicated by severe comorbidities. **Materials and Methods:** Our study included 219 patients with pulmonary multidrug-resistant TB (MDR-TB) who were treated in several clinics in St. Petersburg, Russian Federation. Of these patients, 47.0% had extensively drug-resistant TB (XDR-TB), and 48.4% had severe comorbidities. Univariate and multivariate exploratory analyses were performed to hypothesize factors affecting treatment success. **Results:** Both extensive drug resistance (XDR-TB) and the presence of comorbidity were significantly associated with a lower probability of successful treatment: OR = 0.56 (CI: 0.32–0.96, *p* = 0.033) and OR = 0.53 (CI: 0.30–0.91, *p* = 0.020), respectively. The use of bedaquiline was significantly associated with successful treatment in cases of XDR-TB: OR = 4.15 (CI: 1.32–16.20, *p* = 0.012). Only an insignificant opposite effect was identified for cases of non-XDR-TB: OR = 0.77 (*p* = 0.62). Resistance to thioamides was associated with unsuccessful treatment in cases complicated by comorbidity: OR = 0.46 (CI: 0.21–0.99, *p* = 0.044). Again, an only insignificant opposite effect was identified for cases without comorbidities: OR = 1.11 (*p* = 0.81). Almost all the patterns described above were replicated in the multivariate model. The following two differences with the univariate results were observed. First, the association between the use of bedaquiline and successful treatment became even more pronounced, and, as before, this was true only for XDR-TB: OR = 6.51 (CI: 1.98–26.04, *p* = 0.0036) for XDR-TB, and OR = 0.99 (*p* = 0.98) for non-XDR-TB. Second, the impact of comorbidities on treatment success remained significant only in conjunction with thioamide resistance. In addition, we found that the association between resistance to thioamides and unsuccessful treatment was especially pronounced in cases complicated by heart disease: OR = 0 (CI: 0–0.79, *p* = 0.0088). **Conclusions:** We confirmed that both XDR-TB and the presence of comorbidities are serious challenges in the treatment of tuberculosis. We also have reason to hypothesize that, first, bedaquiline can be a much more crucial component of therapy in cases of XDR-TB than in other cases of MDR-TB and, second, thioamides can play a positive role in cases complicated by comorbidities, especially by heart diseases. These findings should be considered as weak hypotheses that require further verification using independent data, as our analysis was exploratory rather than confirmatory.

## 1. Introduction

Tuberculosis (TB) is one of the major infectious diseases and a leading cause of death worldwide. According to the World Health Organization (WHO) report [1], nearly 10.8 million incident cases of TB were detected in 2023 (a 3.8% increase from 10.4 million cases in 2021), and TB probably returned to being the world’s leading cause of death from a single infectious agent, following three years in which it was replaced by COVID-19. Currently, there are two major closely related challenges in the TB treatment: a large number of cases of drug-resistant TB, as well as cases complicated by severe comorbidities.

Concerning the first challenge, the rise in antimicrobial resistance and the resulting shortage of effective antibiotics are a global public health problem [1,2]. Inadequate use of broad-spectrum antibiotics promotes the spread of multidrug-resistant pathogens [2,3]. According to the WHO report [1], about 400,000 people (95% uncertainty interval [UI]: 360,000–440,000) with multidrug-resistant or rifampicin-resistant TB (MDR-TB/RR-TB) were identified worldwide in 2023. Unfortunately, the treatment success rate for patients with drug-resistant TB remains low and does not exceed 60–68% globally among those who started treatment in 2019–2021 [1]. To treat drug-resistant TB (DR-TB), second-line anti-TB drugs and their combinations are used [1,2,3,4,5,6]. A key drug in modern treatment regimens is bedaquiline, which belongs to the group of diarylquinolines, a new class of anti-tuberculosis compounds [7,8,9,10,11,12,13]. The bactericidal effect of bedaquiline results from the specific inhibition of the proton pump of mycobacterial adenosine-5′-triphosphate (ATP) synthase, which disrupts energy production and ultimately leads to microbial cell death [7,8]. In new treatment regimens, bedaquiline is combined with other drugs, such as pretomanid and linezolid [14,15,16].

Concerning the second challenge, comorbidities significantly impact TB development, treatment outcomes, and patient prognosis [17,18,19,20,21,22,23,24,25,26,27,28]. Cases of drug-resistant TB are of particular concern [27]. There is a complex relationship between TB and various comorbidities, such as diabetes, depression, HIV, and other chronic conditions, which complicate TB management [22,23,24,25,26,27] and may increase the risk of drug resistance [28]. In general, the efficacy of treatment of any pathology in patients with comorbidities depends on careful consideration of drug interactions, altered pharmacokinetics, disease synergies and individualized dosing strategies [29].

The main goal of our study is to explore how various conditions and drugs interact. Our results address both challenges discussed above. Among other findings, we demonstrated that in cases of extensively drug-resistant TB (XDR-TB), the inclusion of bedaquiline in a treatment regimen may be associated with much higher treatment efficacy than in other cases of MDR-TB. We also found grounds to hypothesize that resistance to thioamides is particularly detrimental in cases complicated by comorbidities, especially heart disease. → It is important to note that the results presented should be regarded as tentative hypotheses that require further verification with independent data, as our analysis was exploratory rather than confirmatory [30,31].

## 2. Materials and Methods

### 2.1. Patients

Our study included 219 patients with pulmonary MDR-TB who were treated in several TB clinics in St. Petersburg, Russian Federation. According to the study design, male and female patients with MDR-TB confirmed by bacteriological methods were included. Exclusion criteria were as follows: age under 18 years, pregnancy or breastfeeding, HIV infection (due to the need for antiviral therapy and variability in the level of immunosuppression among patients with HIV), generalized tuberculosis, absence of chemotherapy, requirement for surgical treatment at the time of enrollment, and lack of patient consent to participate. All patients underwent a comprehensive examination to further assess treatment success based on clinical, bacteriological excretion, and X-ray data. Outcomes “cured” or “treatment completed” were considered as success [32], and other outcomes were considered as non-success. General characteristics of patients are presented in Table 1. The analyzed dataset can be found in the Supplementary Materials (file “mdr_tb_data.xlsx”).

**Figure 1:**
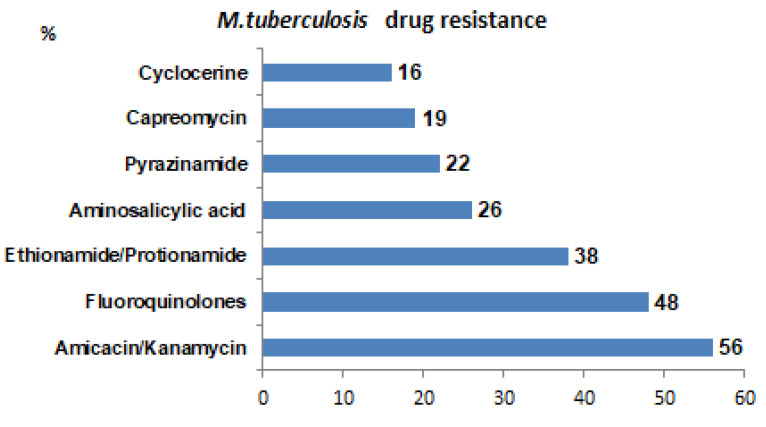
*M. tuberculosis* drug resistance for patients with MDR-TB. For each drug, the percentage of patients with resistance to it is indicated. The most common resistances are to amikacin / kanamycin, fluoroquinolones, and thioamides (%).

**Figure 2:**
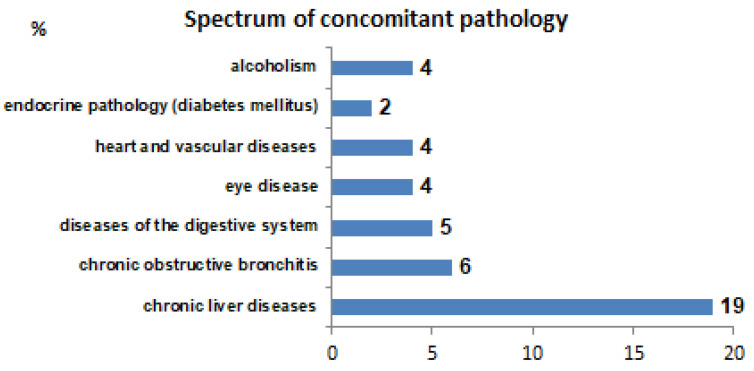
Comorbidities in patients with MDR-TB. For each comorbidity (%), the percentage of patients with it is indicated. Diseases of the digestive system include stomach and duodenal ulcer, chronic gastritis, cholecystitis, and pancreatitis. Eye diseases include myopia, hypermetropia, and cataracts. Heart diseases mainly include coronary heart disease. The most common comorbidity in the sample is Hepatitis C.

The drug susceptibility spectrum of *M. tuberculosis* was determined in all patients. Frequencies of resistance to second-line drugs are presented in Figure 1. Frequencies of various comorbidities in the sample studied are presented in Figure 2.

All patients with pulmonary TB were prescribed a therapeutic regime taking into account the data on drug susceptibility of the pathogen, body weight, and concomitant pathology in accordance with the existing domestic and international recommendations for the treatment of pulmonary MDR-TB and XDR-TB [1,2,16,33]. Therapeutic regimes included various combinations of the following drugs that was prescribed based on the drug susceptibility spectrum of *M. tuberculosis*: ethambutol, pyrazinamide, kanamycin, amikacin, capreomycin, fluoroquinolones, prothionamide, ethionamide, cycloserine, terizidone, aminosalicylic acid, linezolid, amoxicillin, clavulanic acid, imipenem, cilastatin, meropenem, bedaquiline, and thioureidoiminomethylpyridinium perchlorate.

We used the Charlson Comorbidity Index (CCI), which is the most widely used oncology score for assessing comorbid status and can also be applied in the context of TB [26,27]. It assigns weights to comorbidities as follows: 1 point for myocardial infarction, heart failure, cerebrovascular disease, chronic non-specific lung disease, connective tissue disease, peptic ulcer disease, mild liver damage, or diabetes mellitus; 2 points for hemiplegia, moderate or severe kidney damage, diabetes mellitus with organ or system damage, malignant neoplasms, leukemia, or lymphoma; 3 points for moderate or severe liver damage; 6 points for oncopathology with metastases or HIV infection (AIDS stage); and 1 point for every ten years of life in patients over 40.

### 2.2. Statistical Analysis

The following key features were included in the dataset for analysis: gender, age category, CCI, details on comorbidities (binary indicators for certain types of comorbidities), drug resistance type (XDR-TB or only MDR-TB), drug resistance details (binary indicators for each drug), bedaquiline usage, and treatment outcome. The dataset structure is described in detail in the legend (see file “mdr_tb_data.xlsx” in the Supplementary Materials). All features were numerically encoded and used in univariate and multivariate models in binarized form. Data analysis was conducted using the R software environment version 4.2.1 [34].

Concerning the data preparation, we note the following. The absence of discrepancies was verified between the type of drug resistance (XDR-TB or only MDR-TB) and resistances to individual drugs specified for each patient, as well as between CCI and the individual types of comorbidities. Namely, we monitored the consistency of data with the formal definitions of XDR-TB and CCI. In the columns involved in the analysis, there were very few missing values (4 cells in the entire dataset). They were automatically removed by the R functions used for the analysis. Outliers were also not a concern because all the data was binary or categorical.

In the case of small data, the correct choice of goodness-of-fit test may be important when testing hypotheses about the impact of features on the outcome. A comprehensive study and final recommendations for the selection of goodness-of-fit test for small samples can be found in [35]. Following these recommendations in the univariate analysis of the dataset in question always led to the choice in favor of the ‘N−1’ chi-squared test. The R code containing the selection of the criterion according to [35] and the implementation of the ‘N−1’ chi-squared test can be found in the Supplementary Materials (file “Campbell_tests.r”). The epitools package [36] for the R software environment was also used for univariate analysis: the mid-*p* method [37] was generally applied to calculate univariate odds ratios (OR) and their confidence intervals (conditional maximum likelihood estimation was once applied for an extremal case of OR = 0).

For multivariate analysis, logistic regression was used. Multivariate analysis allows for considering data with dependencies between features. However, situations where one feature becomes significant only in the presence of another binary feature require a special approach. In univariate analysis, we accounted for such situations by examining corresponding sub-samples, while in multivariate analysis, we considered composite variables obtained from the two features in question by using the logical AND operator.

In univariate analysis, we evaluated the significance of features in the whole sample as well as in some natural sub-samples determined by other features (e.g., patients with XDR-TB or patients with comorbidities). Variables for the multivariate model were chosen in accordance with the univariate results. Namely, if some binary feature [Feature 1] was significant in univariate analysis and a different binary feature [Feature 2] was significant only in the sub-sample where [Feature 1] was true, then the variables [Feature 1] and [Feature 1 AND Feature 2] are used in the multivariate model.

The code in R is contained in Supplementary Materials (file “mdr_tb_analysis.r”).

## 3. Results

First, we summarize that the most common resistant drugs are amikacin/kanamycin, fluoroquinolones, and thioamides (see Figure 1). The most common comorbidity in the sample is Hepatitis C (see Figure 2).

**Figure 3:**
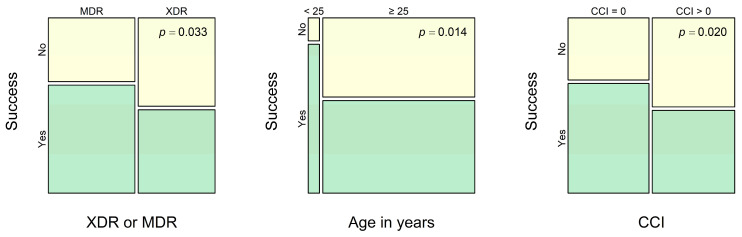
Univariate analysis of MDR-TB treatment efficacy for the entire sample. The success rate differs between XDR-TB cases and other MDR-TB cases, between patients with ages less than 25 years and not less than 25 years, and between cases with CCI = 0 and with CCI > 0. Cases of treatment success are shown in green, and other cases are shown in yellow.

Results of univariate analysis show that both XDR-TB and the presence of comorbidity (in terms of CCI > 0) are significantly associated with a lower probability of successful treatment, while young age (less than 25 years), on the contrary, is associated with a more favorable prognosis (see Figure 3 and Table 2).

**Figure 4:**
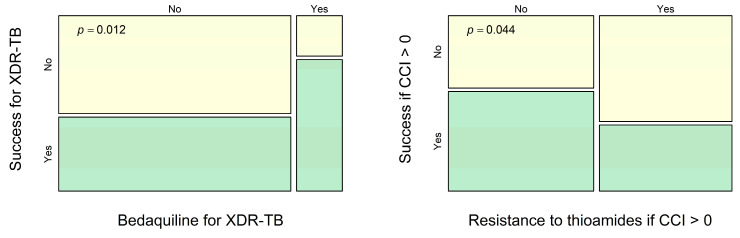
Univariate analysis of MDR-TB treatment efficacy for sub-samples. For patients with XDR-TB, the success rate differs between regimes with bedaquiline and without it. For patients with CCI > 0, the success rate differs between cases with resistance to thioamides and without it. Cases of treatment success are shown in green, and other cases are shown in yellow.

We also see that the use of bedaquiline is significantly associated with successful treatment in cases of XDR-TB (see Figure 4 and Table 2). For the complementary sub-sample of MDR-TB cases that are not XDR-TB cases, we found only an insignificant opposite effect (OR = 0.77, *p* = 0.62), and for the entire sample, the effect was also insignificant (OR = 1.68, *p* = 0.17). Since type II error can be large, this fact does not necessarily mean that bedaquiline does not increase the effectiveness of therapy for non-XDR-TB. However, we have reason to believe that in cases of XDR-TB, bedaquiline can be a much more crucial component of therapy than in other cases of MDR-TB.

Finally, the resistance to thioamides can be associated with unsuccessful treatment in cases complicated by comorbidity (see Figure 4 and Table 2). Again, for the complementary sub-sample of MDR-TB cases with CCI = 0, we found only an insignificant opposite effect (OR = 1.11, *p* = 0.81). For the entire sample, the effect was also insignificant (OR = 0.61, *p* = 0.073). Thus, we have reason to hypothesize that thioamides can play an important role in treating patients with comorbidities.

Almost all the regularities described above were replicated in the multivariate model (see Table 2). The association between the use of bedaquiline and successful treatment became even more pronounced (see Table 2). As before, this association was observed only for XDR-TB. When adding to the model the variable accounting for the effect of bedaquiline in non-XDR-TB cases, we obtained for it OR = 0.99, *p* = 0.98. The same was true for thioamide resistance and comorbidities. Thioamide resistance in cases CCI > 0 remained associated with unsuccessful treatment (see Table 2). However, for the additional variable accounting for thioamide resistance in cases CCI = 0, we had only the insignificant opposite effect with OR = 1.36, *p* = 0.50. It is also worth noting that the impact of comorbidities on treatment success remained significant only in conjunction with thioamide resistance.

**Figure 5:**
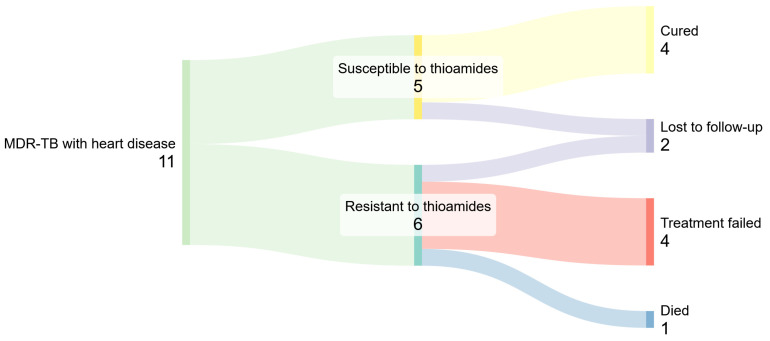
Resistance to thioamides in patients with MDR-TB complicated by heart diseases. For patients with resistance, TB treatment was not successful.

As a final step of our exploratory research, we propose a more specific version of the hypothesis regarding the role of thioamides in the treatment of patients with comorbidities. Specifically, we studied the impact of resistance to thioamides separately across various groups of comorbidities (as previously noted, this factor did not have even an insignificant negative effect in patients without comorbidities). It turned out that a more specific version of the hypothesis could be formulated as follows: in patients with heart diseases, resistance to thioamides may negatively affect TB treatment outcomes. Among 11 patients with heart diseases, 6 were resistant to thioamides. In all 6 cases, treatment was unsuccessful. Of the remaining 5 patients (without thioamide resistance), 4 were cured, and only 1 was lost to follow-up. The corresponding effect size was OR = 0 (CI: 0–0.79, *p* = 0.0088). Further details are shown in Figure 5.

Finally, we note that while multivariate analysis and composite variables can partially solve the problem of non-randomized data, there may be unaccounted confounders such as treatment regimens and adherence. This is one more argument for treating our results only as new weak hypotheses.

## 4. Discussion

Almost half of the patients (47.0%) with MDR-TB had XDR-TB (see Table 1 and Figure 1). On the other hand, 48.4% of patients presented with severe comorbidities (see Table 1 and Figure 2). Both factors significantly complicate treatment selection and are associated with a more severe disease course and a lower probability of successful treatment.

Concerning our new hypotheses, several points should be emphasized. In studies on the efficacy of bedaquiline (see, e.g., the meta-analysis [11]), its absolute efficacy was most often examined (i.e., without comparison with other drugs). Moreover, statistical analyses were typically conducted for all patients with MDR-TB combined. In contrast, our study assessed the relative efficacy of including bedaquiline in treatment regimens compared with its absence, and we performed this analysis separately for patients with XDR-TB and for those with non-XDR MDR-TB. Our results are consistent with study [38], which also suggests that in the absence of fluoroquinolone resistance, the necessity of using bedaquiline may be questioned.

Comparing this study with our previous work on comorbidity [27], it should be noted that although the patient samples substantially overlap, the aims and results were entirely different. Study [27] did not address either the use of bedaquiline or resistance to specific drugs. For the present study, the dataset was refined, which enabled examination of the interactions between drug resistance profiles and comorbidities.

Regarding the specific hypothesis about the role of thioamides in cases complicated by heart disease, it is consistent with the fact that many second-line anti-TB drugs (such as fluoroquinolones, bedaquiline, and linezolid) are cardiotoxic [39,40,41], and resistance to thioamides further reduces the number of suitable treatment options for such patients. It is also worth noting that the WHO does not currently classify thioamides as essential medications, prioritizing the BPaL/BPaLM regimen [16].

As for the limitations of this study, we again emphasize its exploratory nature, which precludes treating the findings as anything more than hypotheses [30,31]. No a priori hypotheses were formulated, and a large number of feature combinations were tested, which increases the likelihood of spurious associations (in such cases, a small *p*-value is not a sufficient criterion). Additionally, the current dataset lacks information on treatment regimens and adherence, preventing adjustment for these critical confounders. Such data could significantly affect the results. In the context of the general discussion, we next consider some comorbidities and drug interactions in more detail.

Diabetes mellitus (DM) is a major comorbidity affecting TB patients globally. The WHO Global Tuberculosis Report 2024 [1] estimates that approximately 400,000 TB cases are associated with diabetes worldwide, a number expected to rise with increasing diabetes prevalence, particularly in high-TB-burden low- and middle-income countries. TB and DM form a vicious cycle: DM impairs immune function (reducing polymorphonuclear leukocyte activity, chemotaxis, and phagocytosis), and hyperglycemia promotes *Mycobacterium tuberculosis* growth. TB-DM patients often present with more severe symptoms, including weight loss, dyspnea, prolonged fever, and more extensive lung lesions (e.g., parenchymal lesions, cavities, and bilateral involvement). Hyperglycemia may reduce anti-TB drug efficacy, and anti-TB drugs can interfere with DM management. TB patients with DM face a higher risk of developing drug resistance [42]. In Kerala, India, while the overall TB treatment success rate was 86.7%, unsuccessful outcomes were most common among patients with diabetes (16.6% of unsuccessful cases) [23]. A Polish study of 19,217 TB patients found that those with comorbidities had lower treatment success rates and higher mortality. DM was associated with an increased risk of TB mortality (OR = 1.9) and a higher risk of death from other causes (OR = 4.5) [22].

Chronic respiratory diseases (particularly asthma) are the second most common comorbidity after diabetes among TB patients in India, present in 19.3% of cases. These conditions can mimic or mask TB symptoms (delaying diagnosis), exacerbate pulmonary symptoms during TB treatment, and require careful management of potential drug interactions between bronchodilators and anti-TB medications [23].

Renal insufficiency necessitates dose adjustments for renally cleared drugs like ethambutol and consideration of hemodialysis effects on drug levels. It also increases the risk of drug-induced liver injury from hepatotoxic TB medications, requiring alternative regimens and close monitoring [43,44].

TB and Hepatitis C (HCV) are two major global infectious diseases, and their coinfection presents significant health challenges. Key concerns include the prevalence of HCV in TB populations [45] and potential drug interactions between anti-TB and anti-HCV medications [46]. Co-treatment of DR-TB and HCV is feasible, with potential benefits including increased treatment success rates and reduced treatment failures for both conditions [47].

Bedaquiline has transformed the treatment of DR-TB since its approval, offering new hope for patients with MDR-TB and XDR-TB [7,8,9,10,11,12,13]. It demonstrates superior efficacy compared to traditional DR-TB regimens in diverse populations, including those with comorbidities such as diabetes. The WHO currently recommends a 6-month BPaLM regimen (bedaquiline, pretomanid, linezolid, moxifloxacin), often combined with fluoroquinolones, linezolid, clofazimine, and cycloserine [14,15,16].

Antagonistic interactions between TB drugs and other medications represent a significant challenge in TB treatment. They potentially lead to reduced therapeutic efficacy, treatment failure, and the development of drug resistance. One well-documented antagonistic interaction involves aspirin (acetylsalicylic acid) and isoniazid. A study in mice showed simultaneous administration of aspirin (20 mg/kg) and isoniazid (25 mg/kg) resulted in incomplete elimination of *M. tuberculosis* from the lungs (2.46 ± 0.30 log10 CFU) compared to complete elimination with isoniazid alone [48].

New clinical data reveal complex interactions between first-line TB drugs. In a cohort of 100 pulmonary TB patients (65% HIV co-infected), the analysis identified negative interactions when isoniazid Cmax was <4.6 mg/L and rifampicin Cmax/MIC was <28. Under these threshold conditions, isoniazid exerted an antagonistic effect on the 2-month sputum culture conversion rate [49].

Antagonistic interactions in TB treatment are a multifaceted problem involving microbiological, pharmacokinetic, and host factors. With TB treatment increasingly applied to patients with complex comorbidities and polypharmacy, awareness of these interactions becomes critical. Strategies such as therapeutic drug monitoring, optimized treatment regimens, and careful drug selection may help mitigate these effects and improve outcomes. Future research should focus on identifying additional clinically significant interactions and developing standardized approaches for managing them in diverse populations.

Finally, while no direct interactions between bedaquiline and thioamides have been reported, their concomitant use requires careful management of overlapping toxicities.

## 5. Conclusions

We once again confirmed that both XDR-TB and the presence of comorbidities are serious challenges in the treatment of tuberculosis. These two factors are significantly associated with a lower probability of successful treatment. We proposed the following two hypotheses concerning them. First, bnot beline is extremely important in XDR-TB cases and may not be so important in other MDR-TB cases. Second, thioamides may be important in cases complicated by comorbidities, especially by heart diseases. These hypotheses require further verification using independent data.

## Figures and Tables

**Table 1:** General characteristics of patients with MDR-TB.

Gender	
Male	160 (73.1%)
Female	59 (26.9%)
Age	
18–24	15 (6.8%)
25–40	105 (47.9%)
41–60	89 (40.6%)
61–80	10 (4.6%)
Conditions	
Patients with XDR-TB	103 (47.0%)
Patients with comorbidities	106 (48.4%)
Treatment outcomes	
Success (cured or treatment completed)	123 (56.2%)
Other outcomes	96 (43.8%)

**Table 2:** Results of univariate and multivariate analysis of MDR-TB treatment efficacy. Statistically significant positive associations with treatment success are highlighted in green, and statistically significant negative associations are highlighted in red.

Features	Univariate Analysis		Multivariate Analysis	
	OR (95% CI)	*p*-Value	OR (95% CI)	*p*-Value
CCI > 0	0.53 (0.30, 0.91)	0.020	0.81 (0.41, 1.62)	0.55
XDR-TB	0.56 (0.32, 0.96)	0.033	0.49 (0.27, 0.88)	0.018
Age less than 25 years	5.20 (1.37, 36.90)	0.014	4.87 (1.24, 32.41)	0.045
Bedaquiline for XDR-TB	4.15 (1.32, 16.20)	0.012	6.51 (1.98, 26.04)	0.0036
Resistance to thioamides if CCI > 0	0.46 (0.21, 0.99)	0.044	0.40 (0.17, 0.89)	0.028

## Data Availability

The dataset and code can be found in the Supplementary Files to the article. Further inquiries can be directed to the corresponding author.

## Supplementary Materials

The following supporting information can be downloaded at: https://www.mdpi.com/article/10.3390/antibiotics14100986/s1, Supporting materials include files Campbell_tests.r, mdr_tb_analysis.r, and mdr_tb_data.xlsx.
